# The Hospital Nursing Supplement

**Published:** 1893-07-29

**Authors:** 


"The Hospital" Nursing Mirror.

Being the Extra Nursing Supplement of "The Hospital" Newspaper.

[Contributions for this Supplement should be addressed to the Editor, The Hospital, 428, Strand, London, W.C., and should have the word "Nursing" plainly written in left-hand top corner of the envelope.]

### News from the Nursing World.

### OUR CHRISTMAS COMPETITIONS.

We anticipate that a large number of garments will this year be made by our readers for the adult-sick poor who have to spend their Christmas day within hospital walls. We find that the parcels which are the result of our annual competition receive such hearty welcomes at the institutions to which they are sent, that we hope to see them increase in bulk and in number each year. The prizes will be: (1) For the best pair of socks knitted by a nurse, 5s.; (2) for the best pair knitted by any Hospital reader, 5s.; for the best made flannel shirt, 10s.; for the best flannel or flannelette bed-jacket, 10s.; for the best flannel petticoat, 10s.; for the best made and simplest shaped dressing gown made and cut out by a nurse, 20s. Long seams may be done by machine.

### UNIFORM VERSUS REGISTRATION.

Great energy characterises the writers who have recently strongly denounced outdoor uniforms for nurses. Whether trained or untrained correspondents, they are alike zealous and somewhat illogical. If a few private soldiers disgraced their uniform, the country would hardly clamour for the army to be divested of its distinctive dress. Moreover, a new "point of vantage," gained by the retention of outdoor uniform, unexpectedly obtrudes itself, and we find to our surprise that some people select their nurses because they admire the shades of their cloaks, bonnets, and veils. A lady says she has seen nurses wearing a certain shade of colour in the parks, and "being much attracted by their appearance, wishes to know how to get one." Therefore, in catering for the public taste, it almost seems as if harmonious clothing were to be put before training, experience, certificates, or any other kind of registration. As for doing away with outdoor uniform, who would dare think of it, when it apparently serves sometimes to secure an unexpected engagement for the wearer.

### IN SERVICE BUT NOT OF SERVICE.

The obvious bad taste of dressing housemaids like hospital nurses seems to be gaining ground. Of course there is a difference in details, but these are unrecognisable by unprofessional observers. Besides being bad taste it is also unfair, for the maid is expected to behave as what she is not, and the nurse is sometimes asked to do what she ought not, at any rate when the uniform is worn in a doctor's house. Small things show which way the wind blows, and an observant person soon learns how to distinguish between the two women, but it is sometimes rather difficult. The other day a lady pointed out to the woman who was opening the door for her to depart, after an interview with a doctor, a piece of cotton wool which had formed part of the dressing of her child's injured hand. "I think it had better be burnt, please." "I'll tell a servant," said the wearer of uniform. "Thank you," said the stranger, and went away resolved that when her child needed attention which she could not give herself, she would engage as attendant a clean and willing maid who would be answerable for the safe disposal of dressings, rather than an anomaly who posed as a parlourmaid.

### AN AWKWARD OMISSION.

Of the daily instances of careless work which come before us, inaccurate addresses, especially as regards numbers, are peculiarly characteristic of a certain class of correspondents. A curious omission occurred recently when invitations were sent out for an evening entertainment and public opening of a new establishment and no address at all appeared on the invitation cards, which set forth that carriages could be ordered at ten p.m. It is difficult to see what object would be gained by the latter proceeding, when their destination might have to be left to chance to discover.

### ARE CURLS COMFORTS?

The wearing of fringes by nurses has been often enough written against. Wise matrons generally decline to receive probationers who cannot conform to a style of hairdressing better suited to a nurse's uniform, the result being that many girls look prettier and younger and better during their training than at any previous or subsequent period of their careers. Some private nurses add fringes to their heads when they adopt coloured stockings and bangles, to the obvious distress and distraction of the sober-minded. If the hair curled naturally it would not so much matter; then it would be merely untidy. But when curling irons have to come into play, they are a patient's most serious rivals, and the time they absorb is generally illspared from duty or from sleep. We hope it is not often that an even more serious accusation can be brought against the fringe wearer, but "A Victim" has recounted an occasion on which she was roused from an early sleep, of which she was sorely in need, by a sudden influx of light. Startled and dismayed, she called out, "Nurse, nurse, what is this glare? It disturbs me." "I cannot possibly see to curl my fringe unless the gas is turned on full," calmly replied the attendant, who had certainly mistaken her vocation.

### CURIOSITIES IN DISEASE.

The illnesses from which paupers suffer are surely of an exceptional character, altogether different from the diseases of the rich. Inmates of workhouse infirmaries are evidently able to reserve their ailments for the daytime, and it is only from sheer obstinacy that a poor old man or woman dies in the night. "Night nurses are not required," has been decreed by many Boards of Guardians. Coventry gives the latest instance of this, but, strange to say, the Medical Officer sides with the Guardians in the matter; and the report of the last meeting tells us that as the two day nurses' salaries had lately been increased the work was being properly done. However, this somewhat illogical statement did not conclude the affair, and sick paupers have yet a chance of being treated humanely by night, as well as by day, for we read in the *Coleshill Advertiser* that "The Clerk said he thought the Local Government Board were anxious to know what the Guardians intended to do about the night nurse before sanctioning the advance." . . . . We may, therefore, understand that the L.G.B. do not mean to allow this important nursing detail to be quietly shelved unless they have proof that the sickness of paupers is of so abnormal a variety that it remains dormant at night!

### POOR ECONOMY.

Everybody is supposed to have some pet economy, and to save in one small way even whilst extravagant in others. One housewife is devoted to cinders, and another to bits of string, whilst a son perhaps develops an attachment to old socks, which his sisters naturally remark is "all very well, but he has not to mend them!" A piece of economy at which, however, no one can smile, is perpetrated by the Oldham Guardians, who carry thrift to the extent of deducting from the holidays of the staff every extra "day off" they have enjoyed during the year. The nominal annual holiday of fourteen days has therefore to be undesirably curtailed, and where a liberal administration would encourage a hard working member of their staff to enjoy an occasional day off duty, the Oldham Guardians aim rather at a system of "All work and no play," which threatens to make of "Jack" a very "dull boy indeed."

### AN UNDERPAID AMATEUR.

Untrained attendants are naturally remunerated at a lower rate than certificated nurses, but this fact does not explain the recent behaviour of the Guardians at Garstang. They installed a young woman in charge of a man and his wife suffering from small-pox. Being a relation of the patients, it no doubt seemed quite natural for her to give up a comfortable situation for the sake of nursing them and looking after their six children. So she was actually day and night nurse to the sick, and also caretaker to the whole family. At first an allowance of 15s. per week was made her; out of this she had to provide food and firing for the household. She eventually applied to have it increased, and after two hours' discussion the Guardians agreed to raise it to 20s. An arrangement like this will hardly tempt other amateurs to abandon their own regular and profitable occupations to enable the Guardians to escape giving just wages to ensure adequate isolation for cases of infectious diseases.

### FESTIVITIES AT WORKSOP.

The Royal wedding was suitably honoured at Worksop, and of the funds collected for carrying out the festivities, a balance remained in hand when all expenses had been paid. The sum of £14 8s. 2d. was divided between the Worksop Dispensary and the Dispensary Nursing Fund.

### QUEEN'S NURSES.

The Southampton Branch of the Queen Victoria Jubilee Institute is doing excellent work. The Superintendent is in communication with a number of respectable women who are available for night watching and caretaking, under the supervision of the Queen's Nurses. The branch at Southampton was only started in October, when Stannard House, in Bellevue Road, was made a home for a superintendent and two nurses, all thoroughly trained.

### SEA-SHELLS.

How patiently the children pick up shells, and hunt for them in all kinds of holes and corners which their elders overlook. There is something very fascinating about shell-seeking, and it is a pleasant feature in seaside holidays. What becomes of the shells afterwards? They turn up in various places in the course of the year, and the most unsuitable spot in which they can be discovered is an invalid's bed. When kind little people are persevering enough to collect a number of pretty shells for sick children, we are unwilling to slight their offerings, yet the acceptance of them means all kinds of dangers for small patients. They get the dainty shells strewn in their beds, and nurses are driven to despair at the risks to their sensitive skins. Let the healthy holiday-makers go on collecting dozens and hundreds of shells, only they had better give them to invalids who are not confined to bed. Poor little workhouse children, whose pleasures are few, would eagerly welcome the spoils of the sea.

### PROPOSED NURSE-TRAINING IN IRELAND.

The Local Government Board in Ireland is recommending the Guardians to arrange for training probationers in their Workhouse Infirmaries. They also express their willingness to share all reasonable expenses. The ultimate object in training suitable young women in this way is to secure an adequate supply of qualified nurses for the wards, but the immediate aim is merely to give elementary instruction in fever and general nursing, in case an epidemic of cholera should arise. Such a movement requires to be made cautiously, for though it might be possible to give a certain amount of instruction in a very short time to intelligent girls, yet there is serious danger in letting loose on the community persons who have acquired such an elementary acquaintance with the profession of skilled nursing. If a few weeks' residence in the infirmary is considered sufficient preparation for nursing patients in their own homes, the poor must surely have to put up with very unskilful treatment. After enjoying such comparative freedom as is associated with outdoor work, these young women will hardly be contented to return to the subordinate position of probationers, and so go through the proper period of training, after they have enjoyed the triumph of posing as nurses during an epidemic. It would be well if the Guardians of these towns were to put themselves into communication with the Irish Branch of the Q.V.J. Institute in Dublin, and see what advice on the subject is given by persons who understand the proper organisation of district nursing.

### SHORT ITEMS.

In the August number of *Nursing Notes* the first of a series of articles on "The Nursing of Sick Children" will appear. They are written by Miss C. J. Wood.—The new Convalescent Home at Withernsea for Hull and East Riding had a successful opening ceremony the other day; the place will be of enormous benefit to many invalids. The first party, between thirty and forty in number, took up their residence on 5th inst.—The nursing of the patients at the North-Eastern Hospital will be newly arranged for after September 30th, when St. John's House relinquishes the charge.—Bishop's Stortford is to have a cottage hospital; the land will be the gift of Sir Walter Gilbey, and the building and furnishing are undertaken by Mrs. Bartle Frere and some friends, as a memorial to the late Mr. Bartle Frere. The managers of the Royal Infirmary, Edinburgh, have decided to continue to admit women students.

### Sanitation for Nurses.

By a Sanitary Inspector.

### XV.—APPARATUS FOR DISINFECTION.

The apparatus usually employed for disinfecting by steam is the "Washington Lyon's Patent Steam Disinfector." Dr. Parsons gives those conditions which are necessary in a disinfecting chamber or apparatus: first, the heat must be uniformly distributed throughout the interior; second, it must be possible to maintain a constant temperature during the whole period of disinfection; third, there must be facilities for ascertaining what is the actual temperature of the interior at any time whilst disinfection is going on. In all the steam disinfecting apparatuses all these conditions are fulfilled, but this is not always the case with the dry heat apparatuses, and it is especially difficult to attain an evenly distributed heat throughout the interior.

The Washington Lyon's Disinfector consists of a large very strong interior iron chamber, oval in shape; its walls are double, with a space between, made of what is known as "boiler plate," a specially strong form of iron plate. At either end is a separate chamber to receive the articles before and after the process, and each of these chambers has a door, tightly fitting, and shutting against india-rubber; it is hinged, and is readily opened and shut in spite of its weight, as it is made to run on a curved rail. One door is used for the introduction into the chamber of the articles to be disinfected, and by the other door they are removed after the process is complete. Two pipes lead from the boiler into the apparatus, one communicating with the space between the double walls or "jacket," and the other with the interior of the chamber, and each is provided with a safety valve and a gauge to indicate the exact pressure of the steam within, the ordinary pressure being five pounds to the square inch in the chamber, and ten lbs. in the "jacket." There is also a pipe to let the steam out of both the chamber and the jacket after the process, and another provided to carry off the water, so that it may not accumulate. The pressure gauge also indicates the temperature in the interior chamber by means of a dial and needle. When the apparatus is to be used, steam is first turned on into the jacket, which warms the walls of the interior chamber, whereby the condensation of the steam when turned on in the interior chamber will be prevented, and also there will be no loss of heat. The articles to be disinfected are placed in the wire cage, or hung on the racks, and these are pushed in on rails through the inlet door, and this is then firmly screwed down. It is recommended to leave these articles then in the inner chamber, the steam circulating only in the "jacket" till they become hot. Attention to this point will prevent condensation, and will reduce the wetting of the articles to a minimum. Then the steam is admitted into the interior chamber till the gauge indicates that the proper temperature has been attained, and this is continued for half an hour, or longer, according to the character of the articles being disinfected. At the conclusion the steam is turned off, first from the interior chamber, but allowed to circulate still in the jacket, which will help to dry the articles. Then the outlet door is opened, and the articles are drawn out into the outer chamber, and in a very short time the articles are perfectly dry without any trouble or assistance; in fact, the articles are never more than very slightly damped by the steam. This is the apparatus used in most, if not all, disinfecting stations. A temperature of 250 deg. F. is readily obtained, and much higher temperatures can be easily got by simply superheating the steam in the interior chamber. This is done by increasing the pressure in the jacket, but not that in the chamber.

When the disinfection is to be accomplished by hot, dry air, the apparatus generally used is the "Nottingham Self regulating Disinfecting Apparatus," invented by Dr. Ransome. This consists of an iron chamber, cubical in form, encased in wood, with a thick layer of felt interposed between the wood and the iron. At each end of the chamber there is a double door. The heat is generated by means of a ring of gas burners enclosed in an iron tube, and the heated air, mixed with the various products of combustion, is carried along a flue placed horizontally, and so led into the chamber which it enters by perforated holes in the floor, the object being to diffuse the heat equally throughout the chamber. The method of regulating the heat is very ingenious. The flue contains the bulb of a thermometer, and a self-acting mercurial regulator through which the gas has to pass as it goes to supply the burners. This regulator is so arranged that as the temperature of the chamber increases the mercury expands and encroaches on the slit through which the gas has to pass, and by narrowing this slit, first reduces and then cuts off the supply of gas to the burners. An outlet flue controlled by a valve, and furnished with a thermometer, is placed at the top of the chamber, and in connection with this outlet there is an arrangement to extinguish fire in case the articles should take fire. This is done by means of a link of fusible metal, so that when the temperature exceeds 300 deg. F. the metal melts and shoots down a damper, which cuts off the supply of gas. This apparatus is heated by currents of hot air, a method which has been found to produce a more uniform temperature throughout the interior than when heated by radiant heat, that is, by applying heat direct from burning coals or gas to the floor and sides. In the latter method the walls and floor of the chamber get very hot, and any articles coming in contact with them are scorched; whereas when the chamber is heated by previously warmed air, the floor and walls are never hotter than the air itself.

The apparatus generally used for disinfecting by moist hot air is "Bradford's Patent Disinfecting Apparatus." It is composed of two parts, the one called a "container," the other the heating apparatus. The container is a large box made of iron and rectangular in shape, and covered with a non-conducting material, open below, and hung up by chains, &c., from pillars, very much like a gas-holder. The roof is provided with an adjustable ventilator and a thermometer, the bulb of which is in the interior and the stem outside, so that the temperature can be easily read. The heating apparatus consists of three long compartments, placed side by side. The central one contains the fuel, placed on a fire-waggon, and the two side ones are for ventilation, and communicate with the fire-chamber by means of side valves. The fire-chamber is provided with a door at one end and with a flue. The waggon which holds the fire can be run in or out on rails. The best fuel for this apparatus is peat, but coke, charcoal, or coal can also be used. A galvanised iron rack is placed over the fire-chamber, and on this are placed the articles of clothing to be disinfected. The air of the chamber is kept moist by means of a large shallow vessel of water placed at the bottom of the container and over the fire-chamber. The container rests on a bed of sand, so as to prevent any loss of heat. The whole thing is on wheels, and can be readily moved from place to place. The advantage of dry heat is that it does not produce shrinking in woollen materials, nor does it felt flannel. Moreover, so long as dry heat is not great enough to scorch the articles, it does not alter the colour of those which are dyed, whereas steam or boiling water will cause all but "fast" colours to run. However, these trifling advantages of dry heat cannot be set against the far greater efficiency of steam as a dis infectant.

### Educational Standards for Nurses.

By Miss Isabel A. Hampton, Superintendent of the Johns Hopkins Hospital, Baltimore, Maryland.

### III.—WORK TO BE ACCOMPLISHED IN THE FUTURE.

It may seem that I find little that is good in the system at present followed in our training schools. I am far from wishing to disparage pioneer efforts, but I would maintain that now that our oldest school in America has attained to its majority, we can no longer fall back upon the plea that our art is still in its infancy. Our founders achieved well and nobly, but it surely was not intended that we should work on forever on the old lines. There are plenty of problems to work out, and schools for nurses are capable of much finer work than has yet been done if we, in whose hands the work is now entrusted, are willing to take a broad and comprehensive view of the subject. The principal of a school for nurses performs the least part of her duty, and throws away many of her privileges, if she is content to confine herself to the limitations of her own particular school. She must look into and go abroad among other schools and teach her nurses to do the same, recognizing what is good in others and being ever ready to adopt any improvement. There is much that we can learn from each other, and sooner or later we must also recognize the fact that we are all trained nurses, and that until something of a common standard is reached, the imperfections of the few must be borne by all. Briefly then, some of our chief aims should be to bring about a spirit of unity among the various schools, to establish a standard of education upon which we may all be judged, and which will, of necessity, be the result of no individual mind or committee, but the consensus of the impartial judgments of many really experienced in the requirements for such work, for in this way alone can we command a thoroughness of work and a selection of women that we cannot now boast of. In doing this the first step should be to bring about in all our schools as far as possible a uniform system of instruction, so that the requirements for graduation should be about the same in each. We might advisedly lengthen the course of instruction in training schools to three years, with eight hours a day of practical work. This would relieve the hospital and school of having to deal with so much new material at so frequent intervals. It would then be possible to select our nurses much more carefully than can sometimes be done, for it happens at times that vacancies occur which must be filled without waiting for the right candidates to present themselves, and it would insure far better results by securing to the hospital nurses with more practical experience. A school naturally divides itself into two classes—the quite competent, and those who are fairly competent. The first division is apt, bright, intelligent, and readily taught; and at the end of the second year one is unwilling to part with them just yet, seeing in them the superintendents and good hospital workers of the future, and the opportunity should be given them of developing their executive ability. Then the incompetent ones are dull and slow of comprehension, and they require to be taught over and over again, and at the end of two years glimmers of the fact that they are beginning to be really interested in the work for the work's sake may be discerned, and one is very loth to let them go until the interest becomes a reality; and so for them really the third year is needed before they may be safely trusted to their own devices.

### Advantages of the Three Years' Course.

The three years' course would also tend to exclude the purely commercial woman who enters the school and gets through the course with as little exertion to herself as possible, only seeing in everything she does future dollars and cents, and never working for the love of her work. With this length of course it would be possible to make a better sub-division of the pupils into classes, so that every member could receive practical teaching, her class teaching and lectures according to her grade in the school and her individual ability. There should be stated times for entrance into the school, and the teaching year should be divided according to the academic terms usually adopted in our public schools and colleges. To see a class of students trying to listen to a lecture on a hot July night is barbarous. The summer months should be reserved for vacations and practical nursing only. In short, the educational atmosphere should be encouraged and developed in every way possible. Even the name of "Nurses' Home" should be changed to "Nurses' School," as the accepted term "Home," as applied to institutions, has no educational significance; and the title of the teacher should be Principal of the School as well as Superintendent of Nurses.

### The Position of Nurses in their Third Year of Training.

In this third year those who are capable should be made head nurses, or some direct responsibility should be laid upon them, and those who wish to fit themselves for teachers in other schools should have the opportunity of acting as assistants to the principal of the school, to learn the clerical and administrative duties of such positions. In this way a class of two might be under her instruction for a year at a time, their positions being those of senior and junior assistants, with a moderate salary attached. Unfortunately few women get these opportunities, and the majority are forced to assume the charge of training schools with no equipment for the work further than that they have been able to acquire as head nurses, and the hospital school, and often the new superintendent herself, suffers accordingly while she is gaining the necessary experience. In fact, a normal school for preparing women for such posts is quite as necessary as those established for other kinds of teachers. The eight-hour system will also be advisable, for the reasons that the health of the nurses will not bear the strain of a three-years' course with longer hours; besides, is it not poor economy and mistaken judgment for a country to sacrifice the health of one class of people in trying to restore the health of others? Then it would do away with the continual breaks in the day's work caused by the half-day and two hours' recreation system, and if a systematic course of theoretical teaching is entered upon shorter hours of practical work are absolutely necessary, as the overpowering physical weariness following a long day's work makes mental effort out of the question, and to require tired-out women to attend evening lectures after nine hours' physical exertion, and the no little mental excitement attendant upon hospital work, is little short of tyranny. And this mental development is necessary for the best results in the work, and if we would command an intelligent class of women.

### Cottage and Smaller Hospitals.

In consideration of education for nurses we must not overlook the smaller hospitals, cottage hospitals, &c., for they have their work to do as well as the large institutions, but that they are in no position to offer adequate teaching of experience to a woman who would become a thorough nurse is very evident. How, then, can we meet the problem of supplying good nursing, and at the same time making good nurses? It can only be met by the larger schools entering into arrangements with the smaller schools to supplement their teaching. This plan, of course, would require that the standard of women and of education should be the same, and the teaching on practically the same basis, while the head nurses of the smaller schools must be thoroughly competent women. In a city where distances are not too great, one school may successfully undertake the care of two or three hospitals. For, instance, a children's hospital may better be associated with a general training school, and the same holds good with a hospital dealing with obstetrics or with any other special branch. As for private sanatoriums owned by private individuals, the nursing should unquestionably be done by salaried nurses who have graduated from some reputable school.

PRACTICAL QUALIFICATIONS OF CANDIDATES.

A final word as to the practical qualifications which should be required of women who present themselves as candidates to be taught nursing. A good practical education should be insisted upon. By this is meant the final examinations in the best high schools in the country, special stress being laid upon the candidate's ability to express herself either in writing or orally with quickness and accuracy. Her knowledge of arithmetic should be of an eminently practical nature, and so that she can readily deal with problems involving fractions, percentage, book-keeping, &c. This much is absolutely necessary. Of course, more than this is desirable, as no other study develope the reasoning powers in the same practical way, and women who do not possess any education in arithmetic beyond the few simple rules simply applied are at once placed in a disadvantageous position upon entering upon their work in a modern hospital. Of course, if she has in addition a knowledge of languages and a broad general reading the candidate is all the better prepared for undertaking and obtaining success in her career as a nurse. Aside from this mental equipment there are other qualifications of a practical nature that should be insisted upon. Every woman before entering upon hospital work should be a thoroughly trained housekeeper. Practical household economy should be a part of her home education, for in hospital wards the nurses are the stewards, the caretakers of the hospital property, and upon their thrift and careful ordering must depend the economical outlay of the hospital funds. I cannot dwell upon this practical household economy with too great emphasis, for experience has shown me, to a painful extent, how this branch of woman's work is neglected or superficially understood by so many women in all ranks of life. A total lack of, or appreciation for, the principles that govern such work will inevitably be followed by a deficiency in thoroughness and system. In the nurse should be found evidence of this practical knowledge; it should be seen in the way she cares for her own room, her personal appearance, and in the order and system which attends any work to which she puts her hand, and her knowledge of the value of the articles she has to work with should be shown by the way she cares for them. But too often, training schools are obliged to not only teach in two short years all that pertains to nursing, but try as well to teach the first principles at least of domestic science, and much valuable time is spent in doing this that should really be given to nursing. When a graduate goes into a private family and earns the just reproach of being extravagant and careless in the care of the property, and when the details of her work are without finish, the blame should be put down to her early home training and not to her training as a nurse. Space does not permit me to more than touch upon some of the most glaring defects in our system of nursing, and to outline very briefly some changes that might be of general advantage, but I trust that sufficient has been said to arouse interest enough among hospital and training school workers to induce them to persevere in working out the problems, the solution of which will give us more united work, and a more uniform standard of education for all who are to go out into the world as trained nurses; for only from institutions in which the head, the heart, and the hand are trained to work together in harmony can come forth the true nurse.

### Wants and Workers.

THE KYRLE SOCIETY appeals for gifts of flags, such as those used in the decoration of private houses at the time of the Royal wedding. The society lends out flags for Industrial Exhibitions, &c., but has not nearly enough to meet the demands made for these decorations.

### Our American Letter.

BY A TRAINED NURSE.

THE International Nursing Congress has proved a great success, and we cannot but wish that a few of the ladies who have done so much in other countries to promote systematic training could have been present. Miss A. Hughes, of the Metropolitan and National Nursing Association of England, came over to read a paper on the Work of Queen Victoria's Jubilee Institute, and both she and her subject had an appreciative reception. Mrs. Dacre Craven (Miss Florence Lees), also of England, contributed a valuable paper on District Nursing, which appears in our American magazine, *The Trained Nurse*, in the July number.

A paper on the Royal National Pension Fund was contributed by Miss Gordon, the Matron of St Thomas's Hospital and the Nightingale Training School, England; and other interesting subjects were ably treated by other heads of institutions.

Matrons and Superintendents of Nurses who do their duty by their schools can rarely secure sufficient leisure to prosecute prolonged journeyings, and this explains, if it cannot console us, for the absence from the Congress of many British workers whose names are well known in America.

The lecture delivered by Miss Hampton on the Standard of Education for Nurses will be read with interest in all parts of the world; and we feel that Miss Irene Sutliffe's charming paper on Training Schools in America will command equal attention.

Of the fifteen graduates who recently received their diplomas at the Buffalo State Hospital, four were men. We may, therefore, point out to our English sisters that here we have, to a certain extent, overcome the difficulty which seems to beset them over there, in procuring regular training for male nurses. We hear from time to time of the progress made in England with regard to that most necessary branch of nursing, the care of the insane, and from our own side of the water we can report a recent arrangement which enables the pupils of the New Jersey School to study the nursing of nervous diseases at the Blackwood County Insane Asylum.

The new building which has been added to the Brooklyn Hospital, Raymond Street, was thrown open to the inspection of visitors last month, and many hundreds of citizens visited this fine annex. It is finished in the most admirable fashion, and, of course, with all the latest sanitary methods.

We are pleased to hear that there is every prospect of a Training School for Nurses being shortly established in Holyoke, Mass., in connection with the City Hospital, which has not long been opened and is already full.

Nineteen pupils have graduated at the Long Island City Training School, and were each presented, at the conclusion of their two years' course, with a gold medal. In giving these, Professor Dr. McCorkle remarked that they were not only to serve as badges of honour for two years' hard work, but "as a reminder that should misfortune overtake any of them, they would always find a home and a welcome in the College Hospital, where they would receive the care and attention they had been taught to bestow upon others."

### Presentations.

ON leaving the Braintree Branch of the St. Alban's Diocesan Nursing Institution to take a similar position at Bournemouth, Miss Maguire received a purse containing £35 subscribed by 300 friends of various classes. Miss Maguire has worked at Braintree for eleven years, and her services have been warmly appreciated by the numerous sufferers to whom she has ministered during that time.

MISS BLUMENTHAL having resigned her position as Matron to the Fulham Union Infirmary, was presented by the officers of the institution with a handsome gold watch bearing a suitable inscription. Miss Blumenthal also received a set of silver saltcellars from two of her nurses, and an excellent testimonial from the Guardians, by whom her departure is much regretted.

### Everybody's Opinion.

HOSPITALS FOR SOLDIERS' WIVES.

"Calpo" writes: Can anyone kindly give me information about hospitals for soldiers' wives and children in England? Where should one apply for an appointment?

A HOLIDAY IN CORNWALL.

"A Nurse" writes: Can any reader of The Hospital tell me of a Home in Penzance or any other part of Cornwall where I could live economically during my holiday? Fine scenery and steamboat trips would be thoroughly appreciated.

AUSTRALIA OR NEW ZEALAND?

"Sister Y." writes: Would some of your readers give me information as to some place in Greater Britain where a consumptive would be benefited? Also, is it better to go in August or September? Where is a warm, dry climate to be found? Is there any difficulty in getting good medical advice? Is it necessary to take a large quantity of underclothing? All information on this subject will be most gratefully received.

### Novelties for Nurses.

CANFIELD WATERPROOF SPECIALITIES.

We have been supplied with specimens of this excellent fabric, which consists of a layer of rubber between finely-woven stockinette. The result is a delightfully soft and warm material the reverse of the repellant and chilly rubber waterproof substances commonly used. The Canfield waterproof is suitably utilised in a number of ways. It can be most advantageously used in the nursery. Children are no longer liable to the danger of a chill where Canfield sheets are used. They do not so easily spoil in or out of use. The sheets cost from 2s. 6d. upwards. Bibs and pilches, for both of which this is an excellent material, can be had for a small sum. Being absorbent, warm, and durable, we believe that the Canfield rubber specialities will continue to advance in popularity as they undoubtedly deserve.

### Appointments.

Stockton Hospital.—Mrs. Beatly has been made Matron of this hospital. Mrs. Beatly was trained at the Dublin Infirmary, and was afterwards Matron of a private hospital in connection with the infirmary.

Chesterfield and North Derbyshire Hospital.—Miss E. G. Waldron has been appointed Matron of this hospital. She was trained at Leicester Infirmary, and afterwards held an appointment there as Ward Sister for eight years, and recently acted temporarily as store and house keeper. She has our good wishes in her present appointment.

Worcester City and County Institution for Nurses.—Mrs. Mary L. Carroll has been appointed Lady Superintendent of this institution. She was trained at the Nightingale School and by the Metropolitan and National Nursing Association. Mrs. Carroll has been Superintendent of the M.N.N.A. Home at Paddington; Sister at St. Thomas' Hospital; Matron of the County Hospital, Lincoln; and Lady Superintendent of the St. Helena Home. Her large experience renders her specially fitted for her present post, and her testimonials are excellent. We wish her success in her new work.

### Minor Appointments.

Wolverhampton Union.—Miss Anna Menon, who was trained at the Whitechapel Union Infirmary, has been made Superintendent Nurse at the Wolverhampton Union. She was previously staff nurse at Walsall Hospital.

West Ham Hospital.—Miss Margaret Seller has been appointed "Night Sister" at the West Ham Hospital, Stratford, E. Miss Seller trained at the Oldham Infirmary where she remained three years, and was afterwards district nurse at Bredbury, near Stockport.

### The Royal British Nurses' Association.

(From Our Oxford Correspondent.)

The annual meeting of this Association was held at Oxford on July 24th. The proceedings consisted of a meeting in Balliol Hall, with Sir Henry Acland as Chairman, a public luncheon in the hall of Magdalen College, presided over by Her Royal Highness the Princess Christian, who is President of the Association, various social functions in the afternoon, and a visit to the University Museum at six o'clock. About one hundred people attended the meeting. In addition to the Chairman, Dr. Wingfield, the city medical officer, represented the local medical faculty. A number of ladies, one or two college dignitaries, and the visitors from a distance made up the attendance.

The actual business was not very great. The annual report, and a statement of accounts showing a small credit balance, were adopted.

On the proposition of Dr. Bedford Fenwick a resolution was agreed to conveying to the President the "heartfelt gratitude which the British nurses owe to Her Royal Highness for her great efforts to bring about an improvement in their profession," and it was resolved to have the resolution suitably engrossed for presentation to the distinguished lady president

The Chairman, Sir H. Acland, delivered an amicable address, in which he spoke in a general way of the advances made in nursing and of the duties, necessity of proper training, and advantages of corporate action. It was stated that there are at present 2,591 members of the Association, but to what extent the subscriptions have been paid or are still outstanding was not mentioned; 1,932 names were said to be on the list of the Association; Miss Grace Gordon had been appointed lady honorary secretary, and Miss Daisy Robins secretary. The Association "notes with gratification the distinguished position which has been occupied by two of its members, Mrs. Bedford Fenwick and Miss de la Pledge, as delegates to the World's Fair at Chicago." A branch of the Association has been founded in Scotland, with headquarters at Edinburgh. The foundation of a branch in Ireland has been postponed on account of "existing political anxiety." After referring to the badge and to the cholera roll, the report stated that the Association has held and intends to hold classes at which nurses may receive a kind of "post-graduate instruction."

Two pensions have been assigned to disabled nurses, and a "large proportion of the limited income has been spent in pecuniary grants to disabled nurses." The Council make a strong appeal to the charitable for funds for such purposes.

The great event of the year for the Association was its incorporation. Its ex-council congratulate themselves that so far as the charter differs from the draft which was submitted in the petition, it "enlarged the powers prayed for, and conferred others which had not been sought." Also, and to this the Association attaches great importance, the nurses inscribed on the list maintained by the Association were described as being "so registered." Why this is important is not apparent, as "registered" is here used as the equivalent of "entered," and in no other sense! "Thus," adds the report, "the Association had gained all it sought, and more."

A strong recommendation was made that all nurses who "enjoy the distinction" of being enrolled should describe themselves on all suitable occasions as "registered nurses," but nothing was said as to the moral and legal consequences of any such action on their part in the absence of a chartered register.

### Press Notices.

A MEDICAL LAYMAN.

Medicus writes, with reference to Dr. Senn's speech on proposing the toast of the foreign medical guests at the banquet by the Practitioners Club in Chicago (reported in the *British Medical Journal* of July 15th), that while it was right in a technical sense to describe Mr. H. C. Burdett as a layman, yet if a complete medical education at two recognised hospitals, a twenty-five years' practical devotion to the larger medical work of the British Empire and the civilised world, an intense sympathy with, and loyal devotion to, his medical first love, and the production of medical—that is to say, hospital—works of unparalleled magnitude and importance, entitle a man to be considered in the large and unconventional sense as a medical man, then Mr. Burdett is improperly described as a layman.—*British Medical Journal.*

### A Novel Competition for Nurses.

This competition has excited a good deal of interest in various countries, and also in the press. The *British Medical Journal* of last week publishes a full account of it, and there is every prospect that all who have sufficient knowledge, experience, and capacity will enter their names for the competition. It would be of immense advantage if each training school and all institutions which have nursing homes were to arrange to select a representative to compete. Much useful instruction might be given by concerting measures, which would enable all who were interested in the question in each nursing home to combine to elaborate plans, and to enumerate each item which a model nurses' home and training school should contain. No doubt there are many architects who would gladly co-operate with the superintendents and nurses by preparing them the necessary plans to a proper scale free of cost, especially as the name of the architect would be attached to the plans, and full credit would be given to them in the competition. One superintendent has raised four important questions, which we will answer in order.

### Limit as to Area of Buildings.

*Question* 1.—Is there any limit as to the area the building is to cover?

The area must of course be dependent upon the available sites, as well as upon the amount of accommodation required, which will vary according to the size of the home. No limit has been fixed for the purposes of the competition, because it is desired to ascertain to what extent superintendents and nurses, and the architects who may assist them, are alive to the importance of abundance of light and air in every portion of a nurses' home and training school. In towns the circumstances of the district in which the hospital is situated must necessarily govern to a great extent the limit as to the area. In the suburban and rural districts it will be assumed that the managers of the institution will be able to secure a good site. Competitors would therefore be well advised to state their views on this question as affecting (*a*) town, and (*b*) rural and suburban sites.

### Supplemental Buildings.

*Question* 2.—If attached to a hospital, should the building include laundry, engine-house, &c.?

We desire to give the competitors wide discretion on this point. The circumstances of a modern hospital ought to be such as to free the nurses' home from the necessity of having a laundry or engine-house buildings. It may not always be possible to do without them in the case of old-established institutions, so that each competitor might usefully give a brief expression of their views and experience on this point

### Hospital and Private Nurses.

*Question* 3.—Is the nurses' home for the accommodation of nurses working in a hospital only, or to include a private nursing staff?

In the particulars of the competition, which we published in our issue of the 15th inst., page clvii., we stated that the modern nurses' home and training school should be so designed as to enable it to be attached to a hospital, or under separate management. This question elaborates the point a little, and competitors would be well advised to give their views and reasons for favouring a plan which provides a home (*a*) for the hospital staff only, and (*b*) one for the hospital and private nursing staff together. Existing institutions have erected homes on both these plans, the choice being governed by economical and local considerations to a large extent. It will be obvious that the inclusion of a private nursing staff must necessitate special provision for isolation, disinfection, and separate accommodation in certain cases.

### Expenses of Carrying out Plans.

*Question* 4.—Will the expense of carrying out the plans be taken into consideration?

We are not sure whether we understand this question rightly. If it relates to the cost of erecting a home and training school on the plans submitted, the answer is, yes. Each competitor would be well advised to give an estimate of cost, and to state upon what method it has been worked out.

We shall be glad to answer any further questions, and certainly hope that in the result many excellent plans will be forthcoming, and that everyone who has ideas and experience will not fail to enter for the competition. The amounts of the prizes, viz., £15 (75 dols.) for the first prize, and £5 (25 dols.) for the second prize, have been fixed in the hope that they may stimulate all to do their best.

### For Reading to the Sick.

### "MUTUAL LOVE."

We do not often think of God's love to us, partly, perhaps, because we do not recognise it in the numberless mercies and blessings which he showers upon us every day and all through our lives. If we did realise it, I think we should try to return it in some measure, for our Lord has told us in the most solemn words, which we dare not despise, that to love God and our brethren is the most important thing in the world. The one love hangs on the other, as St. John, the beloved disciple, says, "He that loveth God will love his brother also."

Illness is the time in which we should properly examine ourselves as to the state of our hearts towards God, for sickness shows us the uncertainty of life, and if we should be called on to meet our God, how could we do it hopefully unless we love Him? His promise is, "I will love them that love Me." We may not hate God—few could do that—but it is not enough to be indifferent to Him; we must love Him with all our hearts, with all our minds, with all our souls, and with all our strength, and show it by worshipping Him, and honouring His holy Name and His Word, and serving Him truly all the days of our life.

And lest anyone should ask, "How can we love God whom we have not seen?" He has joined us in one great brotherhood, and said, "Inasmuch as ye shall have been loving and kind and considerate unto one of the least of these little ones, ye will have done it unto Me." All that would appear mean and worthless to us, all who are soiled and worn with sin, we must not despise, for our mutual share in Jesus's blood imparts an everlasting bond of holiest brotherhood. This tie of love is an immense help in keeping men together and preventing discord and disunion. The Queen, in her letter of thanks to her subjects for the kindness and warmth with which they had commemorated the marriage of her grandson, the Duke of York, took from it a cheerful view of the future and the stability of her kingdom, for she very touchingly and truly said, "My love to my people and their love to me are the great bond of strength to my Empire." And this is true also of spiritual things, God's love for us and our love for Him strengthen Christ's Kingdom upon earth; for let the temptations, the persecutions, the afflictions of the saints be ever so great, yet with mutual love the Church of Christ shall dwell in the unity of the spirit, and the bond of peace and the gates of hell shall not prevail against it. We can get this love by keeping our eyes fixed on our heavenly Father's perfections, on His wisdom, His mercy, His kindness, His ceaseless care for us (He broods over us as a hen over her chickens), and by imitating Him in our behaviour to our fellow creatures. Our Creator gives and forgives, we may do good and love, and by soft endearments lighten the load of daily life.

Then draw we nearer day by day,
Each to his brethren, all to God;
Let the world take us as she may,
We must not change our road;
but we must remain
Fixed to hold love's banner fast,
And by submission win at last.

### The Muses' Looking Glass.

THE REAL THING AND OTHER TALES. By HENRY JAMES. (Macmillan and Co.)

No one, of course, would go to Mr. Henry James in order to forget his trouble over a rousing plot. Nor perhaps can he be recommended as a feather bed for a tired brain. There is always something of a psychological problem underlying his simplest sketches. His characters never forget they are bound to mean something every time they open their lips, and in looking for the meaning the plain reader is apt at times to grow nervous. Are they obscure or is he dense? He is modestly inclined to the latter opinion, and if his head be aching he lays the volume aside with a sigh. But in some moods and to many persons this perpetual demand on the reader for perception of fine shades of mental development is grateful and stimulating, and more than atones for the complete absence of the ordinary "variety" incidents which form the stock-in-trade of the magazine short story.

The "Real Thing" is a delicate bit of satire on a very familiar aspect of British respectability. Major and Mrs. Monarch, reduced to the point of acting as models for an artist in black and white, are models in more senses than one. "Their good looks had been their capital, and they had good-humouredly made the most of the career that this resource marked out for them. It was in their faces, the blankness, the deep intellectual repose of the twenty years of country house visiting which had given them pleasant intonations. I could see the sunny drawing-rooms, sprinkled with periodicals she didn't read, in which Mrs. Monarch had continuously sat. I could see the wet shrubberies in which she had walked, equipped to admiration for either exercise. I could see the rich covers the Major had helped to shoot, and the wonderful garments in which, late at night, he repaired to the smoking-room to talk about them. They didn't do anything themselves, but they were welcome. They looked so well everywhere; they gratified the general relish for stature, complexion, and 'form.' They knew it without fatuity or vulgarity, and they respected themselves in consequence. They were not superficial; they were thorough, and kept themselves up—it had been their line. People with such a taste for activity had to have some line. I could feel how in a dull house they could have been counted upon for cheerfulness. At present something had happened—it didn't matter what; their little income had grown less; it had grown least—and they had to do something for pocket money."

It was natural that they should feel sure of figuring to perfection in the illustrations of a fashionable novel, and that the artist with some misgivings should be persuaded to try. The history of the failure is told with much sympathetic insight. Of the two Major Monarch is the more distinct personality. "To listen to him was to combine the excitement of going out with the economy of staying at home. There was only one hindrance: that I seemed not to know any of the people he and his wife had known. I think he wondered extremely during the term of our intercourse whom the deuce I *did* know. He hadn't a stray sixpence of an idea to fumble for, so we didn't spin it very fine; we confined ourselves to questions of leather and even of liquor (saddlery and breeches makers and how to get good claret cheap), and matters like "good trains," and the habits of small game." Every one has met a Major Monarch, and found him pleasant company. Of Mrs. Monarchs, too, there are great plenty, but they are generally associated, perhaps, with well stifled yawns, echoing the experience of the artist to whom she sat. "At first I was extremely pleased with her lady-like air . . . but after a few times I began to find her too insurmountably stiff. . . . I placed her in every conceivable position, but she managed to obliterate their differences. She was always a lady, certainly, and into the bargain, was always the same lady. She was the real thing, but always the same thing. There were moments when I was oppressed by the serenity of her confidence that she was the real thing."

The rival model is "meagre little Miss Churm." "She couldn't spell and she loved beer, but she had two or three points, and practice, and a knack, and a kind of whimsical sensibility, and a love of the theatre, and seven sisters, and not an ounce of respect, especially for the 'h.'"

Alas, Mrs. Monarch was not "in it" in comparison with this freckled cockney who could represent everything, from a fine lady to a shepherdess. There is a humorous little scene where the "real thing" and the imitation article are brought into contact, not altogether to the advantage of the former. Miss Churm arrived wet through from Kilburn.

"'I'm all in a soak. There *was* a mess of people in the 'bus. I wish you lived near a stytion,' said Miss Churm. I requested her to get ready as quickly as possible, and she passed into the room in which she always changed her dress. But before going out she asked me what she was to get into this time.

"'It's the Russian Princess, don't you know?' I answered, 'the one with the "golden eyes" in black velvet, for the long thing in the Cheapside.' 'Golden eyes? I *say*!' cried Miss Churm, while my companions watched her with intensity as she withdrew. 'Do you think she looks like a Russian Princess?' Major Monarch asked with lurking alarm. 'When I make her, yes.' 'Oh, if you have to *make* her'—he reasoned acutely. 'That's the most you can ask. There are so many that are not makeable.' 'Well, now, here's a lady,' and with a persuasive smile he passed his arm into his wife's, 'who's already made.' 'Oh, I'm not a Russian Princess,' Mrs. Monarch protested, a little coldly. I could see that she had known some and didn't like them. There immediately was a complication of a kind that I never had to fear with Miss Churm. This young lady came back in black velvet—the gown was rather rusty and very low on her lean shoulders—and with a Japanese fan in her red hands. I reminded her that in the scene I was doing she had to look over someone's head. 'I forget whose it is, but it doesn't matter. Just look over a head.' 'I'd rather look over a stove,' said Miss Churm; and she took her station near the fire. She fell into position, settled herself into a tall attitude, gave a certain backward indication to her head and a certain forward droop to her fan, and looked, at least to my prejudiced sense, distinguished and charming, foreign and dangerous. We left her looking so, while I went downstairs with Major and Mrs. Monarch."

There is something charmingly pathetic in the attitude of the pair when their inevitable failure is brought home to them. They had aspired to represent the hero and heroine of a new novel which the artist was illustrating, and they find their places occupied by the despised Miss Churm and an Italian orange-monger picked out of the street, to do double duty as model and servant. They stand and watch in silence. "Presently I heard Mrs. Monarch's sweet voice beside, or rather above me: 'I wish her hair was a little better done.' I looked up, and she was staring with a strange fixedness at Miss Churm, whose back was turned to her. 'Do you mind me just touching it?' she went on—a question which made me spring up for an instant, as with the instinctive fear that she might do the young lady a harm. But she quieted me with a glance I shall never forget—I confess I should like to have been able to paint *that*, and went for a moment to my model. She spoke to her softly, laying a hand upon her shoulder and bending over her; and as the girl, understanding, gratefully assented, she disposed her rough curls, with a few quick passes, in such a way as to make Miss Churm's head twice as charming. It was one of the most heroic personal services I have ever seen rendered." We part from the pair after all with regret.

### Notes and Queries.

### Queries.

(161) *L.O.S.*—Will anyone kindly give me address of the London Obstetric Society?—*L. S. M.*

(162) *Workhouse Nursing.*—Can you tell me whether the Workhouse Nursing Association has any rules about their nurses having regular nights off duty?—*Rachel.*

(163) *Midwifery.*—Can you tell me of a good book on this subject?—*Nurse Rachel.*

### Answers.

(161) *L O.S. (L. S. M.).*—See answer to Query (68) in THE HOSPITAL of March 11th, 1893.

(162) *Workhouse Nursing (Rachel).*—You should write to the hon. secretary, and ask for the rules of the association.

(163) *Midwifery (Nurse Rachel).*—You should look at our answers to queries, and you will find this one answered fully in current year.

### The Book World for Women and Nurses.

[We invite Correspondence, Criticism, Enquiries, and Notes on Books likely to interest Women and Nurses. Address, Editor, THE HOSPITAL (Nurses' Book World), 428, Strand, W.C.]

HOUSEKEEPING, a guide to domestic management. By Mrs. HUMPHRY. ("Madge," of *Truth.*) F. V. White and Co.

This volume, with the cheerful cover, brims over with most excellent advice to the inexperienced and unwary. Nothing could be better than the hints to a mistress on the management of her cook and kitchen, or the routine which should be followed if the household is to be conducted with dignified orderliness. Where there is no special time fixed for any particular duty, nothing but muddle can ensue, and muddle brings about forgetfulness of stores to be ordered, consequently delay in the preparation of meals, unpunctuality, a constant rush to make up for what has been forgotten, lack of leisure to attend to the niceties of life, impatience on the part of the husband, followed by weary disgust and total annihilation of that happy domestic communion which should be the aim of all good wives. Most wise and just is the recommendation to treat servants with kind consideration, tempered by firmness. It is only fair that those from whom we expect so much should have comfortable rooms and beds, and a sufficiency of good, nourishing food. In some households more care is bestowed on the food and lodging of the horses than on that of the servants. Everyone knows that the aim of stable management is to so feed the animal that the maximum of work may be got out of him with the minimum of wear and tear, and this applies as much to human beings. It comes cheaper in the long run to keep your servants in good health and cheerful spirits by means of comfortable rooms, nutritious food, and a certain amount of relaxation, exercise, and fresh air. They exert themselves far more for a kind and considerate mistress, and do their work with far greater thoroughness. There are also a few judicious words as to the management of husbands. The recipes at the end of the book are good, though we deprecate the slipshod custom of prescribing "a cupful of milk" or "a handful of bread crumbs." It is said elsewhere that men are superior to women as cooks because they invariably weigh or measure their ingredients according to the recognised standards. Women guess at the quantities, and the "handfuls," "pinches," "butter the size of a nut," vary as frequently as the size of the teacup. With all the excellencies of the book, we, nevertheless, think it aims at too much. It reads rather like the "Answers to Correspondents" in a ladies' journal—"The Home," "Cookery," "Dress," "Medicine and Toilet," "Etiquette and Good Form." Ladies are advised to purchase a white silk dress very much as they should make stock, to be used afterwards for all manner of purposes, and flavoured according to taste. One year it is to be "trimmed with green velvet," the next "done up" with cardinal red satin, the third season with jet, after which it ceases to be fresh enough for evening wear, and should be used only for garden parties and race meetings! Mistresses are bidden instruct their parlour-maids to say "Chartreuse or Benedictine, Madam?" and this is emphasised by a footnote on the "conservatism of despotic butlers who condemn (!) footmen to the time-honoured 'ma'am.'" Is "Madge" aware that the Queen is invariably addressed as "ma'am" by her Court? while "madam" has been merely invented to meet the demand of the "ladies" of Mile End as well as Bayswater "to be treated as such."

### JULY REVIEWS.

Few articles will be read with greater interest this month than the sketches in the *New Review* of the present Duchess of York. They are written in a pleasant and sympathetic style, evidently by those well acquainted with the Princess's private life, and agree in presenting a charming picture of the bride. With a mother so nobly prominent in advancing all wise schemes for benefiting the suffering poor, Princess May could hardly fail of philanthropic instincts, but there is much to show that the interest in her poorer neighbours which she has shown almost since infancy has been no mere following of another's lead. She has shown method, perseverance, and self-sacrifice to an unusual degree in her work for others. In her sympathy for underpaid seamstresses and other women workers she has not shrunk from the study of Blue-books and the mastering of the driest details; she examines herself into the details of all charitable schemes submitted for her support, and has a clear head for facts and figures, which makes her opinion worth having. And the element of personal sympathy is not wanting. "She is always thinking of others and how she can best assist her less fortunate sisters. A suffering child at once commands her sympathy. Out of her income she always sets apart a sum to give to poor children. A crippled boy in a village near White Lodge was dying of consumption. Over and over again Princess May would either drive or walk over to see the little sufferer, and sitting down by the bedside in the cottage, would talk and read to him. Often she carried with her delicacies to keep up his wasting frame. Her last visit to the boy was one day on her way to church, when she knew the end was near. Gently giving him a kiss, she wished him good-bye with tears in her eyes. I could tell of many actions of a similar kind, but this one will suffice to show her tenderheartedness and sympathetic nature."

### PUBLICATIONS FOR THE MONTH.

The July number of Cassell's Family Magazine contains an article on some "Royal Princes and Their Brides," illustrated by portraits contemporary with the weddings, which will be read with interest. Fiction is well represented, the two serials are well written, and deal with adventures of an exciting nature. Some idea of the unceasing labour which falls to the lot of public men may be gained from "A Day in the Life of a Cabinet Minister," an account of an interview with the Right Hon. H. H. Fowler, M.P., President of the Local Government Board. Useful information is given in a short article, "A Little Feverish," by "A Family Doctor," who endeavours to combat the erroneous ideas entertained by the multitude on the subject of the temperature of the human body. He tells us that "most people fancy their temperature varies considerably according to their surroundings, and look surprised when they are told that when working hard on a hot summer day their bodily temperature does not differ appreciably from what it is when they are shivering on the coldest day in winter." Those who wish to know how easily they may beautify their rooms at small expense should read "New Lamps for Old," where they will find some really excellent suggestions for the construction of "cosy corners" and home-made bookshelves, work-tables, &c. The idea of utilising old bedposts for standard lamps will commend itself at once to all who possess such relics of the past. Miss Ethel Cuthell contributes a descriptive sketch of the picturesque little village of Studland in the Isle of Purbeck, between Poole and Swanage, a charmingly quiet spot, as yet unspoilt by "trippers." Here is to be found a quaint old church of early Norman architecture.

